# Index-based FT-IR assessment of chrysotile detectability and environmental durability in asbestos cement materials

**DOI:** 10.1038/s41598-026-49524-w

**Published:** 2026-04-21

**Authors:** Gergely Zoltán Macher, Dóra Beke, András Torma

**Affiliations:** 1 Department of Applied Sustainability, Albert Kázmér Mosonmagyaróvár Faculty of Agricultural and Food Sciences, Széchenyi István University, Egyetem tér 1, Győr, 9026 Hungary; 2 Department of Plant Sciences, Albert Kázmér Mosonmagyaróvár Faculty of Agricultural and Food Sciences, Széchenyi István University, Vár tér 2, Mosonmagyaróvár, 9200 Hungary

**Keywords:** Asbestos cement, Environmental exposure, FT-IR spectroscopy, Environmental durability, Index-based analysis, Engineering, Environmental sciences, Materials science

## Abstract

Despite regulatory restrictions, asbestos cement materials remain widely employed among the population, thereby presenting significant environmental and health risks due to their chrysotile asbestos composition and vulnerability to weathering-induced deterioration. This paper explores the environmental durability of asbestos cement products and the detectability of chrysotile fibres using Fourier Transform Infrared spectroscopy, with a focus on an index-based evaluation. The analysis included both environmentally exposed and non-weathered reference samples, categorised into flat sheets, corrugated sheets, and pipes. Key performance indices were developed to assess spectrum matching accuracy, chrysotile fibre detectability, and durability under environmental stress. Results indicate that environmental exposure was associated with higher chrysotile detectability, likely due to surface degradation and increased dusting potential. These findings offer valuable insights for risk assessment and the development of improved monitoring strategies for asbestos-containing materials in various environments. The paper highlights the importance of comprehensive evaluations to understand the environmental behavior and risk profiles of asbestos cement products, which can inform appropriate management approaches for these materials in different applications and settings.

## Introduction

Asbestos is a naturally occurring mineral^1,2^ that has been widely used in the construction and manufacturing industries^3,4^ for its exceptional thermal and chemical resistance properties^5^. The two main types of asbestos are chrysotile (serpentine) and amphibole, with the latter being less suitable for industrial applications due to its inferior qualities^6–8^. Owing to their advantageous properties, including durability, affordability, and versatility, asbestos-containing materials (abbreviation: ACMs), particularly asbestos cement (abbreviation: AC), have become ubiquitous in a wide range of applications across numerous industries over the years^6,9,10^. AC, in particular, is a composite material composed of cement and asbestos fibres, which has found extensive application in roofing, piping, and other building materials due to its durability and cost-effectiveness^11–13^.

However, the presence of asbestos in this material poses significant health and environmental concerns^14–16^. The use of asbestos has been a subject of significant controversy due to its potential health hazards, with exposure to asbestos fibres linked to various respiratory diseases, including asbestosis, lung cancer, and mesothelioma^17^. Furthermore, the growing awareness of the potential health hazards associated with asbestos exposure has led to increased scrutiny, regulation and ban of its use^16–18^. Asbestos fibres can be released into the environment, contaminating the ambient environmental matrices and ecosystems, during the production, installation, or demolition of AC, leading to potential human exposure and respiratory issues^16^. Efforts have been made to develop alternative cement materials that can replace asbestos while maintaining desirable properties^19,20^.

Despite these regulatory restrictions, the environmental persistence and degradation behavior of AC products have received comparatively less attention. Recent studies have demonstrated that AC materials undergo gradual physicochemical degradation when exposed to natural environmental conditions, including moisture, temperature cycling, ultraviolet irradiation, and atmospheric pollutants^21–23^. These factors induce microstructural damage such as matrix leaching, surface carbonation, and loss of mechanical integrity, which in turn facilitate the partial liberation of embedded chrysotile fibres. The degradation rate and fibre release potential have been shown to depend on the composition of the cement matrix, pH variations, and the degree of carbonation^24–26^. Understanding these processes is essential not only for assessing environmental risks but also for evaluating the detectability and spectral response of asbestos fibres within aged materials.

Notwithstanding, AC products remain ubiquitous in the built environment and continue to be prevalent in urban environments. As cement-based materials age, they can become deteriorated and release asbestos fibres, posing significant health risks. Studies have shown that the weathering and degradation of AC products can lead to the release of asbestos fibres into the environment^20,25,27–29^. Disturbing or damaging AC materials, such as roofing or piping, can have serious consequences for human health and the environment. When these materials are broken, cracked, or otherwise compromised, they can release asbestos fibres into the air^17,30,31^ and water. This is a particular concern for older buildings and infrastructure where AC materials were commonly used^32,33^. The risk is particularly high for individuals involved in the renovation, repair, or demolition of structures containing AC, as they may be exposed to high concentrations of airborne asbestos fibres^34,35^. Moreover, the widespread use of AC in public infrastructure, such as water pipes, poses a significant threat to the general population, as the degradation of these materials can contaminate drinking water supplies^25,36,37^.

From a material science perspective, several authors have investigated the durability of AC under environmental exposure using analytical methods such as X-ray diffraction, thermal analysis, and scanning electron microscopy^24,38,39^. These studies reveal that prolonged weathering leads to a decline in the crystalline order of the cement matrix, the partial amorphization of chrysotile, and the formation of secondary phases such as calcite or gypsum^40,41^. Such transformations modify the infrared spectral response of the material, making FT-IR spectroscopy a promising tool for tracing degradation and fibre detectability in aged AC products. Nevertheless, comprehensive spectral-index-based approaches integrating environmental durability aspects remain scarce in the current literature.

Proper management and disposal of these materials are essential to mitigate the potential exposure and health hazards associated with aging AC products^42^. Raising awareness among the general public about the risks associated with AC is crucial to mitigating the potential harm caused by these materials. Public education campaigns, particularly in communities where AC is prevalent, can help individuals understand the dangers of asbestos exposure and the proper procedures for handling and disposing of these materials^43,44^. Public awareness can help avert potential environmental disputes and legal conflicts. Environmental conflicts often arise when the presence of hazardous materials is not adequately addressed^45^. By raising public awareness and promoting responsible practices for the management of AC, the risk of environmental disputes and litigation can be reduced. Additionally, proactive engagement with local authorities and policymakers can encourage the development and implementation of stricter regulations and enforcement measures to prevent the improper handling and disposal of AC^42^.

The goal of an asbestos-free Europe by 2032 has been articulated in EU policy discussions, notably in the opinion of the European Economic and Social Committee on “Freeing the EU from asbestos”^46–48^. As a result of previous unsuccessful attempts to achieve an asbestos-free environment, the year 2023 was not envisioned as the year to attain complete asbestos-free status, but rather a period to establish the strategic foundations and implement a new regulatory framework. Notwithstanding the alignment of the year 2030 with the United Nations’ Sustainable Development Goals, the EU has determined that the technical complexities and financial obstacles associated with asbestos removal necessitate a more realistic target date of 2032. This extended timeframe affords member states the flexibility to implement the required transition in a gradual and systematic manner. Despite the widespread bans on AC products in developed nations, these materials continue to be extensively used in construction, particularly in developing regions, where they are often the most accessible and affordable option for the impoverished population^20,49–52^ The prevalence of AC products in these communities has become a symbol of social and economic inequality, as the health risks associated with asbestos exposure disproportionately affect the disadvantaged. The societal impacts of asbestos exposure, particularly its disproportionate effects on disadvantaged communities, highlight the urgent need for effective management and mitigation strategies^10,42,43,53^.

In this context, understanding the environmental degradation pathways and durability of AC materials becomes fundamental not only for risk assessment but also for the advancement of reliable detection techniques. Incorporating the concept of degradation into FT-IR-based analysis allows for a more holistic evaluation of asbestos detectability in materials subjected to long-term environmental stressors.

To address these challenges, it is essential to develop reliable methods for identifying and assessing asbestos-containing materials. Fourier Transform Infrared spectroscopy (abbreviation: FT-IR) is a powerful tool for the characterization of asbestos and AC materials^54^. FT-IR analysis can provide valuable information about the chemical composition and molecular structure of AC, including the presence of asbestos fibres, such as chrysotile. The FT-IR spectrum of asbestos can be used to identify the specific vibrational modes of the materials, which can help in the detection and quantification of asbestos content^19,55,56^. The integration of FT-IR analysis with other assessment methods can provide a comprehensive understanding of the risks associated with AC materials and guide the development of effective monitoring and remediation strategies^16,56^.

In the context of the growing concern surrounding the environmental and health hazards posed by AC products, particularly with their widespread use, this research aims to provide a comprehensive, novel approach to assessing the environmental durability of such materials. Previous studies have focused primarily on the identification and quantification of asbestos fibres using various analytical techniques, including FT-IR spectroscopy. However, the integration of FT-IR with an index-based analytical framework has yet to be explored in depth. This paper introduces a new methodology by combining FT-IR spectroscopy with an index-based approach to evaluate the environmental degradation of AC. The importance of this work is further underscored by the need for reliable methods to monitor the degradation of AC products, particularly in regions where these materials are still in widespread use. The results of the analysis could contribute significantly to the development of more effective monitoring and remediation strategies, aiding in the safe management and disposal of asbestos-containing materials.

## Methodology

This paper aims to investigate the detectability of chrysotile asbestos and the environmental durability of AC products using FT-IR spectroscopy. The primary aim of this research is to utilize an index-based methodology to investigate the interrelationships between the individual factors examined in the paper and the risk factors associated with exposure.

### Experimental specimens

Three categories of ACMs samples were examined: corrugated AC-sheets, flat AC-sheets, and AC-pipes. Each category provided two sample groups. Within each set, two additional subgroups were established: five samples exposed to various environmental factors and five samples separated from these influences. Multiple measurement experiments were conducted for each sample to assess the conformity rates with the chrysotile asbestos spectra. The number of successful matches was recorded, and statistical evaluation was based on the top 10 matches. This methodology enabled the evaluation of the impact of environmental exposure on individual product categories compared to their corresponding separated counterparts.

The environmentally exposed specimens were obtained from roofing and piping materials that had been in service for approximately 30–40 years under natural outdoor conditions. These materials were subjected to periodic temperature fluctuations (− 10 °C to + 35 °C), moderate to high relative humidity (50–85%), and airborne pollutants characteristic of urban and industrial environments. Prolonged exposure to rainwater, freeze-thaw cycles, and ultraviolet radiation contributed to surface leaching and microstructural deterioration. In contrast, the non-weathered reference samples represent reference materials of the same age that were never exposed to environmental conditions. These specimens were not installed in service but have been stored indoors under covered and stable conditions since their production. Consequently, they can be considered non-weathered reference samples providing a baseline for comparison with the exposed materials.

The initial set of samples consisted of undulating corrugated AC-sheets. These specimens, estimated to be 30–40 years old, exhibited visible signs of erosion and deterioration due to environmental exposure, as well as various deposits. The labeling of the samples exposed to environmental factors ranged from AC-S-E-1 to AC-S-E-5, with the average value denoted as AC-S-E-A. The labeling of the non-weathered reference samples spanned from AC-S-S-1 to AC-S-S-5, with the mean value designated as AC-S-S-A.

The second set consists of flat AC-sheets with comparable aging, displaying effects from environmental exposure. The labeling of the samples subjected to environmental influences ranges from AC-F-E-1 to AC-F-E-5, with the mean value designated as AC-F-E-A. Similarly, the labeling of the non-weathered reference samples spans from AC-F-S-1 to AC-F-S-5, with the mean value labeled as AC-F-S-A.

The third set comprises AC-pipes with similar attributes to the previous samples. The samples exposed to environmental factors were labeled from AC-P-E-1 to AC-P-E-5, with the average denoted as AC-P-E-A. Correspondingly, the non-weathered reference samples were labeled from AC-P-S-1 to AC-P-S-5, with the mean value designated as AC-P-S-A.

Prior to the analysis, all three sets of samples underwent meticulous cleaning with distilled water and isopropyl alcohol to remove any potential impurities that could influence the findings. Subsequently, the samples were cut into uniform sizes and then encased in a multi-component epoxy resin. This epoxy resin provided protection for the samples during the subsequent polishing and analysis processes.

### Sample preparation

The samples were thoroughly cleaned with distilled water and isopropyl alcohol to remove any impurities that could impact the analysis. They were then encased in epoxy resin and cut into discs measuring 30 × 5 mm. The samples were not pulverized into bulk powder. FTIR measurements were performed on polished epoxy-embedded sections prepared from representative fragments of the asbestos-cement materials. These discs were ground to the desired size and mounted on instrument slides for examination. A 0.3-mm diamond crystal was used to scan specific areas of the samples with infrared rays, generating an absorption map with colored spectra corresponding to the materials in the spectrum library, allowing for component identification. To minimize spectral interference from ambient moisture and CO_2_, background spectra were collected before each measurement. The samples were analysed by FTIR in attenuated total reflectance (abbreviation: ATR) mode with a spectral resolution of 4 cm^−1^, and each spectrum was averaged over 16 scans.

### Characterization of chrysotile by FT-IR spectroscopy

All types of asbestos display strong absorption in the 1200–900 cm^−1^ and 600–300 cm^−1^ infrared ranges. The specific asbestos type can be identified qualitatively by analyzing the spectrum up to 200 cm^−1^. Chrysotile asbestos exhibits distinct differences from amphibole asbestos, as it demonstrates significant double hydroxyl groups at 3693 cm^−1^ and 3648 cm^−1^, which are created between layers of hydroxyl groups situated within the primary silicate layers of the lattice. In the present paper, the FTIR-based index approach was used primarily for chrysotile detectability assessment and comparative evaluation of environmentally induced material alteration. In the absence of calibration against reference standards, the method should not be interpreted as a fully validated tool for absolute quantification of cement and AC. For FT-IR measurements, a PerkinElmer Spectrum 400 spectrometer (PerkinElmer, MA, U.S.) was used in reflection and attenuated total reflectance (ATR) modes. Spectral data acquisition was performed at room temperature under controlled humidity, using a 0.3-mm diamond ATR crystal. The applied pressure on the sample was approximately 100 N to ensure consistent optical contact.

### Developing an integrated index approach

To assess the degree of degradation and environmental impact on the AC samples, an integrated index approach was developed. This approach involved calculating several small indices related to the material’s composition and structural properties. The indices were designed to capture both the detectability and degradation characteristics of chrysotile within the AC matrix. Their formulation was inspired by previously established spectral-ratio and similarity indices applied in mineralogical and materials FT-IR analyses^57–59^ and further refined in this study to evaluate the combined influence of environmental exposure and detectability variability in AC materials. The Spectrum Matching Index (SMI) was calculated as Eq. (1).

1 $$SMI = ~\mathop \sum \limits_{{i = 1}}^{n} E_{i} /n$$

where *E*_*i*_ is the spectrum matching ratio, and *n* is the number of samples.

The Exposed Samples Detectability Index (ESDI) was calculated as Eq. (2).

2 $$ESDI = SMI_{{exposed}} - SMI_{{separated}}$$

where *SMI*_*exposed*_ refers to the spectrum matching ratio of the exposed samples, and *SMI*_*separated*_ refers to the spectrum matching ratio of the non-weathered reference samples.

The Relative Detectability Index (RDI) was calculated as Eq. (3).

3 $$RDI = (SMI_{{exposed}} - SMI_{{separated}} /SMI_{{separated}} ) \times 100$$

where *SMI*_*exposed*_ refers to the spectrum matching ratio of the exposed samples, and *SMI*_*separated*_ refers to the spectrum matching ratio of the non-weathered reference samples.

The Degradation Impact Index (DII) was calculated as Eq. (4).

4 $$DII = \sqrt {\mathop \sum \limits_{{i = 1}}^{n} \left( {E_{i} - SMI_{{exposed}} } \right)^{2} /n}$$

where *SMI*_*separated*_ refers to the spectrum matching ratio of the non-weathered reference samples, *SMI*_*exposed*_ refers to the spectrum matching ratio of the exposed samples and *n* is the number of samples.

The Environmental Impact-Exposure Index (EIEI) was calculated as Eq. (5).

5 $$EIEI = SMI_{{exposed}} /SMI_{{separated}}$$

where *SMI*_*exposed*_ refers to the spectrum matching ratio of the exposed samples, and *SMI*_*separated*_ refers to the spectrum matching ratio of the non-weathered reference samples.

The Critical Detectability Index (CDI) was calculated as Eq. (6).

6 $$CDI = \left( {SMI_{i} /\max \left( {SMI_{{total}} } \right)} \right) \times 100$$

where *SMI*_*i*_ refers to the spectrum matching ratio of the i-th samples, and *SMI*_*total*_ refers to the spectrum matching ratio of the total samples.

The Detectability Variation Index (DVI) was calculated as Eq. (7).

7 $$DVI = Var_{{(E_{{exposed}} )}} - Var_{{\left( {E_{{separated}} } \right)}}$$

where *Var* denotes a signed variability term derived from replicate spectral responses and used to indicate detectability fluctuation, *E*_*exposed*_ is the spectrum matching ratio of exposed samples, *E*_*separated*_ is the spectrum matching ratio of non-weathered reference samples.

The Degradation Exposure Equality Index (DEEI) was calculated as Eq. (8).

8 $$DEEI = \left( {\mathop \sum \limits_{{i = 1}}^{n} \left| {SMI_{{exposed}} - SMI_{{separeted}} } \right|} \right)/n$$

where *SMI*_*exposed*_ refers to the spectrum matching ratio of the exposed samples, and *SMI*_*separated*_ refers to the spectrum matching ratio of the non-weathered reference samples.

The Detection Efficiency Index (DEI) was calculated as Eq. (9).

9 $$DEI = 1 - \left( {{\mathrm{Var}}\left( {E_{i} } \right)/\max \left( {Ei} \right)} \right)$$

where *Var* denotes a signed variability term derived from replicate spectral responses and used to indicate detectability fluctuation, *E*_*i*_ is the spectrum matching ratio of samples.

The Environmental Durability Index (EDI) was calculated as Eq. (10).

10 $$EDI = \left( {SMI_{{exposed}} - SMI_{{separated}} } \right)/{\mathrm{max}}\left( {SMI_{{exposed}} ,~SMI_{{separated}} } \right)$$

where *SMI*_*exposed*_ refers to the spectrum matching ratio of the exposed samples, and *SMI*_*separated*_ refers to the spectrum matching ratio of the non-weathered reference samples.

## Results and discussion

**Fig. 1:**
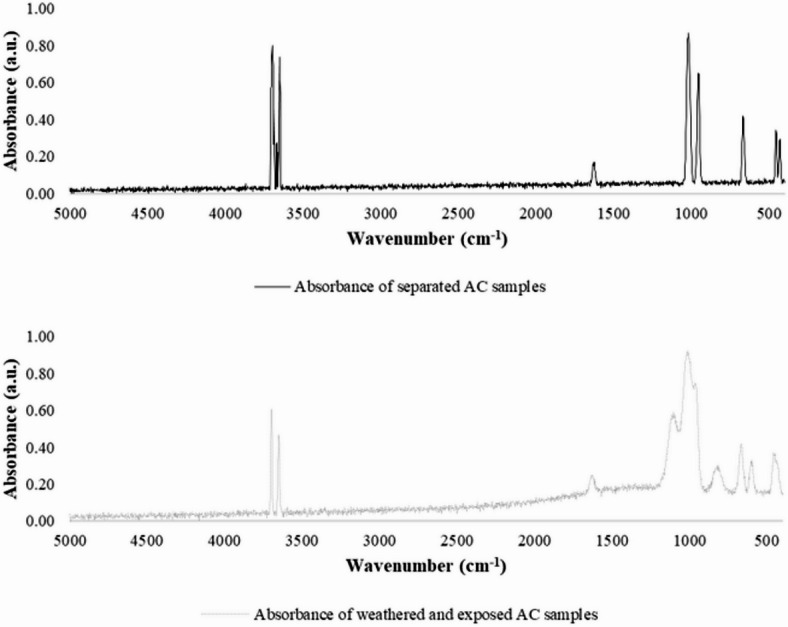
Comparison of averaged absorbance values across all separated and exposed samples.

The FT-IR spectra obtained for both environmentally exposed and non-weathered reference samples are presented in Fig. 1. The characteristic absorption bands of chrysotile were observed at approximately 3693 cm^−1^ and 3648 cm^−1^, corresponding to the inner and interlayer hydroxyl vibrations, while Si–O stretching modes appeared between 960 and 1040 cm^−1^. The exposed AC samples displayed broadened and slightly shifted OH bands, indicating partial dehydroxylation and structural disorder induced by prolonged environmental exposure. These spectral differences substantiate the degradation effects discussed below.

As anticipated, the determination of sub-index values within the framework of the integrated index-based approach yielded results consistent with the expected trends, confirming that FT-IR spectral indicators sensitively reflect both exposure-induced degradation and detectability variations. The findings specifically revealed degradation-related spectral changes and improved chrysotile detectability in AC samples exposed to environmental influences, thereby illustrating the effect of prolonged environmental exposure.

Changes in the Si-O stretching region (900–1100 cm^−1^) were interpreted as indirect spectral signs of matrix alteration that may be associated with increased porosity and fibre liberation. The calculated index values are summarized in Tables 1 and 2, where Table 1 presents sample-specific indices and Table 2 presents pair-based comparative indices for the exposed/reference sample groups. The main reason for the use of AC products in the construction industry is their exceptional durability and resistance to environmental factors^60^. However, not all AC products exhibit the same level of environmental durability. Studies have shown that the durability of these materials is closely linked to their composition and manufacturing processes^20,61^. Certain AC products, such as those with a higher proportion of cement and lower asbestos content, tend to be more resistant to weathering, chemical attacks, and other environmental stressors^62,63^. On the other hand, products with a higher asbestos content or those that have been exposed to specific environmental conditions may be more vulnerable to degradation over time^64^.

**Table 1:**
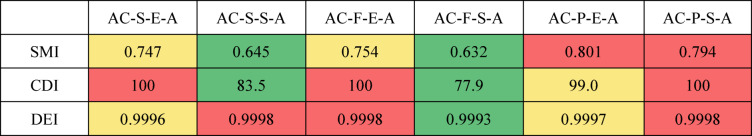
Sample-specific index values for the examined AC sample groups.

AC-asbestos cement, S/F/P-sheet (corrugated)/flat sheet/pipe, E/S-exposed, non-weathered reference, A-average.

**Table 2:**
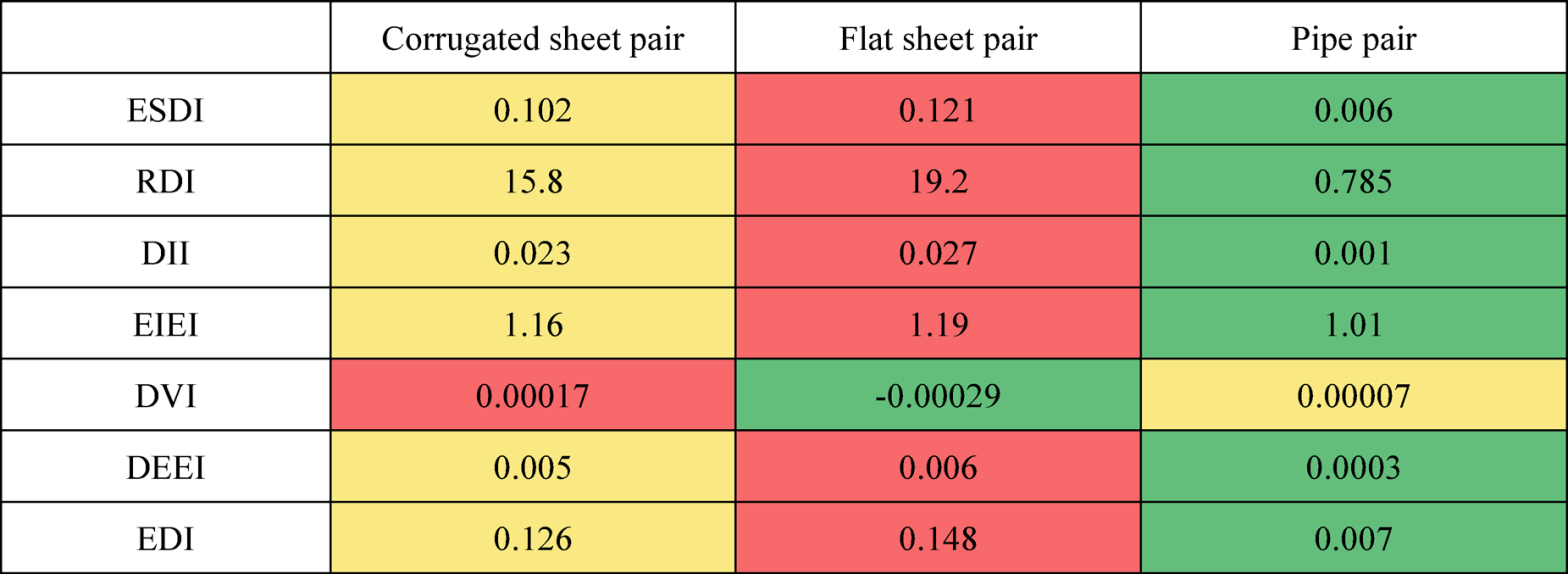
Pair-based comparative index values for exposed and reference AC sample groups.

AC-asbestos cement, S/F/P-sheet (corrugated)/flat sheet/pipe, E/S-exposed, non-weathered reference, A-average.

This observation is consistent with the present findings, where the degradation-related indices (DII and EDI) were higher for samples subjected to environmental influences than for their separated reference counterparts. In other words, the combined and often synergistic nature of the environmental effects can amplify the extent of each degradation parameter, leading to a more pronounced deterioration of the material over time. This aligns with previous reports that emphasize the role of carbonation, microcracking, and leaching processes in the weakening of the cement-asbestos matrix under natural weathering^65^.

The correlation matrix presented in Table 3 provides an exploratory overview of the relationships among the examined indices. Because the analysis was based on a limited number of aggregated group-level observations, the correlation coefficients should be interpreted with caution and should not be regarded as definitive evidence of statistical dependence. Nevertheless, the observed pattern suggests that SMI tends to decrease as degradation-related indices increase, whereas DVI appears to be less closely associated with the other indicators. These trends are useful for preliminary interpretation, but they require validation using larger datasets and sample-level analyses.

**Table 3:**
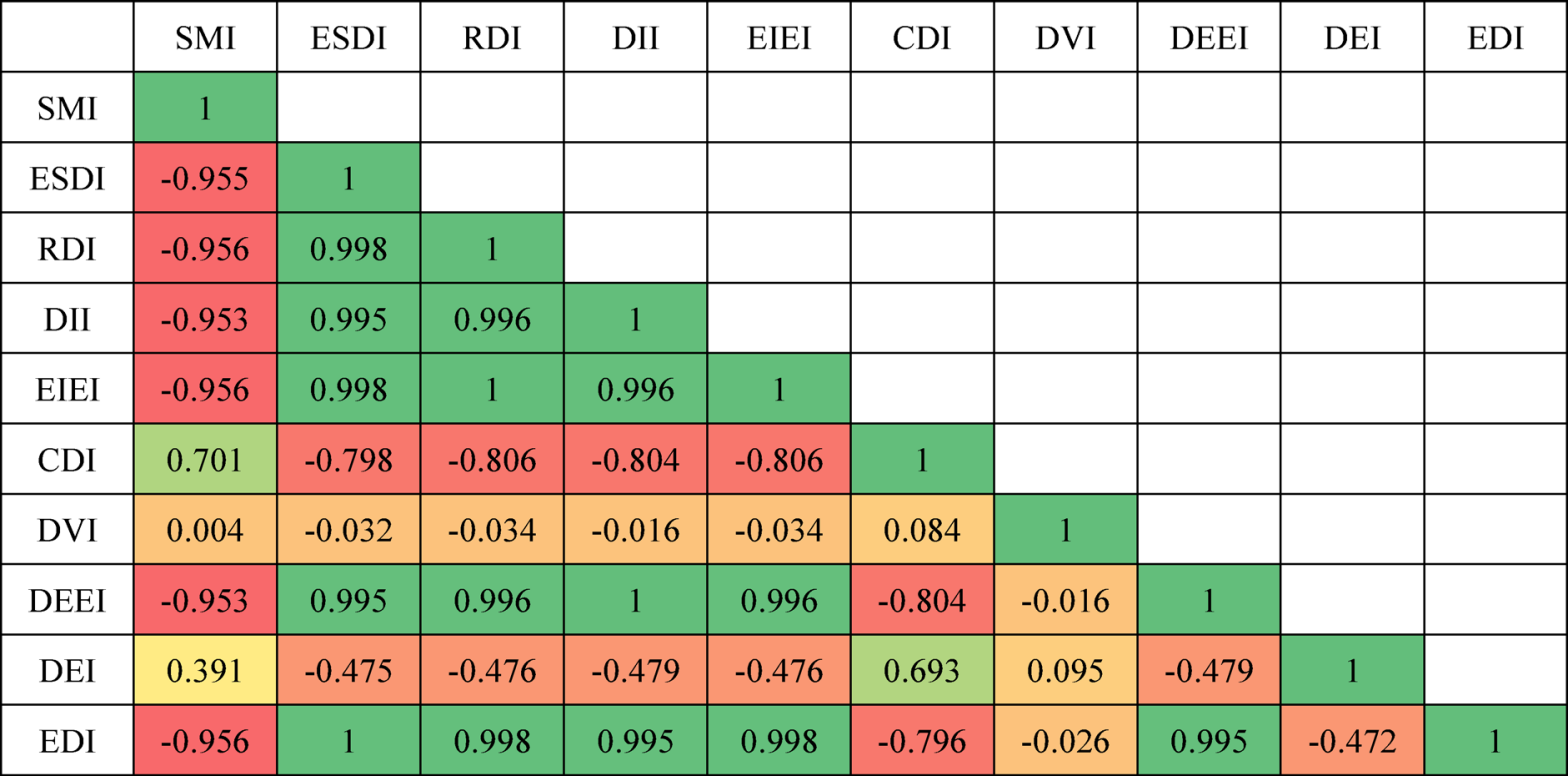
Correlation matrix of the examined index values.

**Fig. 2:**
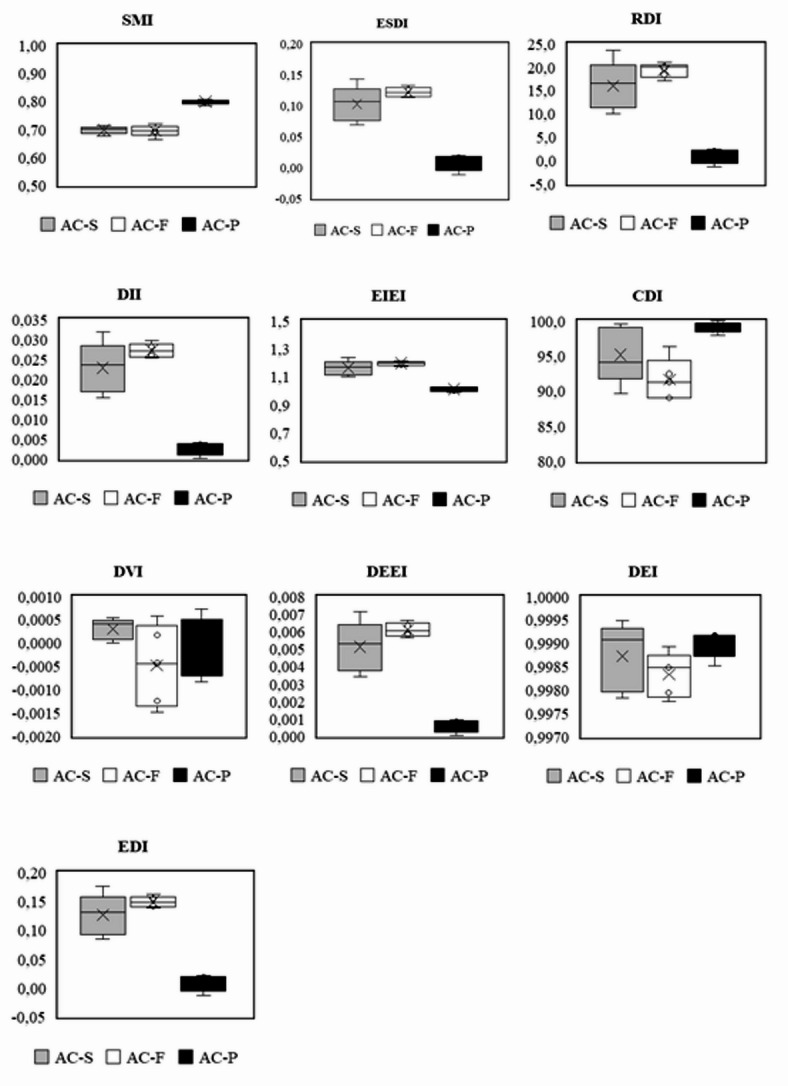
Comparison of index values across different sample groups

The integrated index system proved capable of distinguishing between environmental degradation and detectability effects, providing a quantitative framework that bridges spectral data with material condition assessment. The FT-IR index suite therefore offers a promising diagnostic tool for the non-destructive evaluation of aging AC materials. The index-based testing, approach and quality control of AC materials is particularly important as research in asbestos management and removal is often complex and involves multidimensional problems^66^. AC materials are subject to a number of environmental factors and mechanical impacts over their lifetime, which gradually affect the condition and safety of the material^67^. The index-based approach allows these complex factors to be integrated into a single comprehensive indicator, thus providing a simplified and more effective way of assessing quality control^68,69^. Index-based testing makes it easy to detect variables that could potentially be dangerous and to anticipate problems that could later require significant costs and professional intervention. Future studies should aim to validate the proposed indices against larger datasets and complementary analytical methods such as XRD or SEM, thereby improving the transferability of the approach. Continued refinements and expansions are essential for the index-based assessment and quality control of AC materials, as the present approach represents an initial iteration that requires ongoing enhancement. Furthermore, the metrics require routine updating and maintenance to account for evolving environmental factors, ensuring that future quality assurance of AC products adapts not only to the present but also to the changing industrial and regulatory landscape. This ongoing process of refinement and expansion is crucial for maintaining the accuracy and reliability of the assessment and quality control measures^70^ used for AC materials, which must adapt to the dynamic nature of environmental conditions and industry regulations over time^71^.

More accurate and improved indices can better support safe use and sustainable development in the future, ensuring that risks to public health and the environment are minimised and that the 2032 target is met in a safe, material and cost-optimal way. Index-based assessments, while already delivering significant benefits, can therefore be further strengthened and made more widely applicable in practice through future developments^72^. By expanding the use of these indices and assessments, we can ensure that the use of AC is guided by the best available data and analysis, leading to more informed decision-making and better outcomes for both public health and the environment^73^. The index-based approach, when combined with direct FT-IR spectral validation, provides a scientifically grounded pathway toward data-driven asbestos management, bridging laboratory diagnostics and practical environmental monitoring. The present paper has several limitations. The sample number was limited, the asbestos-cement matrix was heterogeneous, and the FTIR response may have been influenced by surface condition, spectral overlap, and local variability. In the present framework, DVI should be interpreted as a directional variability indicator rather than a strict statistical variance measure. Moreover, the proposed indices were not calibrated against external reference standards, therefore, the approach should be regarded as comparative and semi-quantitative rather than fully quantitative.

## Conclusions

This paper presents an integrated, index-based analytical framework for assessing the environmental durability and detectability of chrysotile fibres in AC products using FT-IR spectroscopy data. The paper categorized the examined products into three main types: flat sheets, corrugated sheets, and pipes. These were exposed to varying degrees of environmental factors, with some samples undergoing direct environmental exposure and others being analyzed under isolated, controlled conditions. To develop the assessment system, key indicators were established to examine the accuracy of spectral matching, the detectability of chrysotile fibres, and the durability changes resulting from environmental stress. The FT-IR spectra of the analyzed samples, particularly in the 3690–3640 cm^−1^ and 960–1040 cm^−1^ regions, confirmed the characteristic absorption features of chrysotile and revealed intensity variations indicative of matrix degradation in environmentally exposed samples.

The results showed that environmental exposure was associated with higher detectability of chrysotile fibres. This observation may be explained by surface degradation and powdering effects, which can facilitate the emergence of fibres at or near the measurement interface and make them more readily detectable. The integration of spectral evidence with the index-based framework therefore provided a comparative means of linking FT-IR signal changes to environmental deterioration processes.

Furthermore, the research results reinforce the necessity to formulate guidelines and policies for handling AC products based on their environmental interactions and long-term performance, considering the increasing demands for diverse application settings and asbestos-free alternatives. Among the main contributions of this paper is the introduction of an integrated set of indices that collectively support the comparative assessment of detectability and degradation-related behaviour in ACMs. These indices were inspired by established spectral-ratio methods used in mineralogical FT-IR studies but were adapted here to evaluate the combined effects of exposure and detectability variability. This paper also clarified the interdependence among degradation-related indices, demonstrating that prolonged exposure leads to measurable shifts in FT-IR parameters, correlating with higher DII and EDI values. These results suggest that the proposed approach is sensitive to differences between exposed and reference (non-weathered) samples, supporting the potential usefulness of the developed indicators.

The findings provide valuable insights into the behavior of these materials under environmental stress and highlight the need for holistic risk assessment and management strategies. The proposed methodology shows potential as a non-destructive analytical approach for comparative monitoring and quality assessment of AC products, in line with the EU’s 2032 asbestos-free objective.

The research showcases a novel, index-based assessment methodology that enables a comparative and semi-quantitative evaluation of the environmental durability and detectability of asbestos fibres in cement-based materials. However, in the absence of calibration against reference standards and validation on larger datasets, the method should be regarded primarily as comparative and semi-quantitative rather than fully quantitative. Future work should aim to validate the developed indices through larger datasets and complementary techniques such as SEM and XRD, further improving the robustness and transferability of the approach.

## Data Availability

The datasets generated during and analysed during the current study are not publicly available but are available from the corresponding author on reasonable request.

## Abbreviations

AC: Asbestos cement
ACM(s): Asbestos-containing material(s)
FT-IR: Fourier-transform infrared spectroscopy
ATR: Attenuated total reflectance
SMI: Spectrum Matching Index
ESDI: Exposed Samples Detectability Index
RDI: Relative Detectability Index
DII: Degradation Impact Index
EIEI: Environmental Impact-Exposure Index
CDI: Critical Detectability Index
DVI: Detectability Variation Index
DEEI: Degradation Exposure Equality Index
DEI: Detection Efficiency Index
EDI: Environmental Durability Index
SEM: Scanning electron microscopy
XRD: X-ray diffraction

## References

[1] Van Orden, D. R. Asbestos. In *Environmental Forensics* 19–33 (Elsevier, 1964). 10.1016/B978-012507751-4/50024-0.

[2] Lee, R. J., Strohmeier, B. R., Bunker, K. L. & Van Orden, D. R. Naturally occurring asbestos—A recurring public policy challenge. *J. Hazard. Mater.* **153**, 1–21. 10.1016/j.jhazmat.2007.11.079 (2008). 18180100 10.1016/j.jhazmat.2007.11.079

[3] Thives, L. P., Ghisi, E., Thives Júnior, J. J. & Vieira, A. S. Is asbestos still a problem in the world? A current review. *J. Environ. Manag.* **319**, 115716. 10.1016/j.jenvman.2022.115716 (2022). 35863303 10.1016/j.jenvman.2022.115716

[4] Durczak, K. et al. Modern methods of asbestos waste management as innovative solutions for recycling and sustainable cement production. *Sustainability* **16**, 8798. 10.3390/su16208798 (2024).

[5] Pawełczyk, A., Božek, F., Grabas, K. & Chęcmanowski, J. Chemical elimination of the harmful properties of asbestos from military facilities. *Waste Manag.* **61**, 377–385. 10.1016/j.wasman.2016.11.041 (2017). 10.1016/j.wasman.2016.11.041 27979425

[6] Shah, N. V., Rao, H. V. B. & Jain, S. C. Investigation on suitability of indigenous asbestos for the manufacture of asbestos cement products. *Trans. Indian Ceram. Soc.* **22**, 109–115. 10.1080/0371750X.1963.10855463 (1963).

[7] Visonà, S. D. et al. Inorganic fiber lung burden in subjects with occupational and/or anthropogenic environmental asbestos exposure in Broni (Pavia, Northern Italy): An SEM-EDS study on autoptic samples. *Int. J. Environ. Res. Public Health* **18**, 2053. 10.3390/ijerph18042053 (2021). 33669843 10.3390/ijerph18042053 PMC7923219

[8] Marzini, L. et al. Asbestos hazard in serpentinite rocks: Influence of mineralogical and structural characteristics on fiber potential release. *Geosciences* **14**, 210. 10.3390/geosciences14080210 (2024).

[9] Bolan, S. et al. Sustainable management of hazardous asbestos-containing materials: Containment, stabilization and inertization. *Sci. Total Environ.* **881**, 163456. 10.1016/j.scitotenv.2023.163456 (2023). 37062308 10.1016/j.scitotenv.2023.163456

[10] Curado, A. et al. The use of asbestos and its consequences: An assessment of environmental impacts and public health risks. *Fibers* **12**, 102. 10.3390/fib12120102 (2024).

[11] Lee, E-S. & Kim, Y-K. Asbestos exposure level and the carcinogenic risk due to corrugated asbestos-cement slate roofs in Korea. *Int. J. Environ. Res. Public Health* **18**, 6925. 10.3390/ijerph18136925 (2021). 34203418 10.3390/ijerph18136925 PMC8297172

[12] Ingham, J. P. Concrete products. In *Geomaterials Under the Microscope* 121–127 (Elsevier, 2013). 10.1016/B978-0-12-407230-5.50014-5.

[13] Brandt, M. J., Johnson, K. M., Elphinston, A. J. & Ratnayaka, D. D. Pipeline design and construction. In *Twort’s Water Supply* 693–742 (Elsevier, 2017). 10.1016/B978-0-08-100025-0.00017-X.

[14] Panzera, T. H., Christoforo, A. L. & Ribeiro Borges, P. H. High performance fibre-reinforced concrete (FRC) for civil engineering applications. In *Advanced Fibre-Reinforced Polymer (FRP) Composites for Structural Applications* 552–581 (Elsevier, 2013). 10.1533/9780857098641.4.552.

[15] Inobeme, A. et al. Asbestos fibres—adverse health and environmental effects and Environmental Protection Agency regulations. In *Technical Organic and Inorganic Fibres from Natural Resources* 647–661 (Elsevier, 2025). 10.1016/B978-0-443-15459-1.00008-5.

[16] Peña-Castro, M., Montero-Acosta, M. & Saba, M. A critical review of asbestos concentrations in water and air, according to exposure sources. *Heliyon* **9**, e15730. 10.1016/j.heliyon.2023.e15730 (2023). 37305461 10.1016/j.heliyon.2023.e15730 PMC10256854

[17] Matthews, J. C. & Stowe, R. Critical data needs associated with asbestos cement pipe renewal methods. *J. Constr. Eng. Manag*. 10.1061/(ASCE)CO.1943-7862.0000914 (2015).

[18] Kwon, J. Impact of naturally occurring asbestos on asbestos ban: Regulations and experience of the Republic of Korea. *Int. J. Environ. Res. Public Health* **19**, 742. 10.3390/ijerph19020742 (2022). 35055562 10.3390/ijerph19020742 PMC8775668

[19] Syahida Adnan, Z., Farhayu Ariffin, N., Maszura Syed Mohsin, S. & Hasanah Abdul Shukor Lim, N. The assessment on the acceptance of waste materials as a partial cement replacement in malaysian construction industry. In *IOP Conference Series: Materials Science and Engineering* Vol. 1092, 012007. 10.1088/1757-899X/1092/1/012007 (2021).

[20] El-Sayed, M., Faheim, A. A., Salman, A. A. & Saleh, A. M. H. Introductory chapter: Cement industry. In *Cement Industry—Optimization, Characterization and Sustainable Application* (IntechOpen, 2021). 10.5772/intechopen.95053.

[21] Mohr, B. J., Biernacki, J. J. & Kurtis, K. E. Supplementary cementitious materials for mitigating degradation of kraft pulp fiber-cement composites. *Cem. Concr. Res.* **37**, 1531–1543. 10.1016/j.cemconres.2007.08.001 (2007).

[22] Tang, L. et al. Experimental investigation on the deterioration of the physical and mechanical properties of autoclaved aerated concrete at elevated temperatures. *High Temp. Mater. Process.*10.1515/htmp-2022-0301 (2024).

[23] Sørensen, M. K. et al. A systematic review of outdoor airborne asbestos concentrations in urban and rural areas. *J. Hazard. Mater. Adv.* **20**, 100926. 10.1016/j.hazadv.2025.100926 (2025).

[24] Butkevics, J. & Atstaja, D. Diversion of asbestos-containing waste from landfilling: Opportunities and challenges. *Sustainability* **17**, 4529. 10.3390/su17104529 (2025).

[25] Zavašnik, J., Šestan, A. & Škapin, S. Degradation of asbestos—Reinforced water supply cement pipes after a long-term operation. *Chemosphere* **287**, 131977. 10.1016/j.chemosphere.2021.131977 (2022). 34454219 10.1016/j.chemosphere.2021.131977

[26] Curado, A. et al. Recovery of end-of-life building materials: Thermal decomposition and phase transformation of chrysotile in asbestos-containing fiber cement boards. *Fibers* **13**, 62. 10.3390/fib13050062 (2025).

[27] Habert, G. Assessing the environmental impact of conventional and ‘green’ cement production. In *Eco-efficient Construction and Building Materials* 199–238 (Elsevier, 2014). 10.1533/9780857097729.2.199.

[28] Zhang, Y-L. et al. Risk assessment of asbestos containing materials in a deteriorated dwelling area using four different methods. *J. Hazard. Mater.* **410**, 124645. 10.1016/j.jhazmat.2020.124645 (2021). 33257124 10.1016/j.jhazmat.2020.124645

[29] Bassani, C. et al. Deterioration status of asbestos-cement roofing sheets assessed by analyzing hyperspectral data. *Remote Sens. Environ.* **109**, 361–378. 10.1016/j.rse.2007.01.014 (2007).

[30] Kim, S. H. et al. Ventilation impairment of residents around a cement plant. *Ann. Occup. Environ. Med.* **27**, 3. 10.1186/s40557-014-0048-6 (2015). 25713724 10.1186/s40557-014-0048-6 PMC4338829

[31] Kottek, M. & Yuen, M. L. Public health risks from asbestos cement roofing. *Am. J. Ind. Med.* **65**, 157–161. 10.1002/ajim.23321 (2022). 34962302 10.1002/ajim.23321 PMC9305126

[32] Singh, R., Vivek, J. M., Rao, B. & Asolekar, S. R. Significance of the presence of asbestos in construction and demolition wastes in India. In *Advances in Waste Management* 303–317 (Springer, 2019). 10.1007/978-981-13-0215-2_21.

[33] Mehra, S., Singh, M., Sharma, G., Kumar, S. & Navishi, Chadha, P. Impact of construction material on environment. In *Ecological and Health Effects of Building Materials* 427–442 (Springer, 2022). 10.1007/978-3-030-76073-1_22.

[34] Kakooel, H. & Normohammadi, M. Asbestos exposure among construction workers during demolition of old houses in Tehran, Iran. *Ind. Health* **52**, 71–77. 10.2486/indhealth.2012-0118 (2014). 24292876 10.2486/indhealth.2012-0118 PMC4202766

[35] Obmiński, A. Pollution of the environment and building interiors during asbestos removal as a result of lack of negative pressure in the working areas. *Sci. Rep.* **14**, 21185. 10.1038/s41598-024-70631-z (2024). 39261502 10.1038/s41598-024-70631-z PMC11391016

[36] Speil, S. & Leineweber, J. P. Asbestos minerals in modern technology. *Environ. Res.* **2**, 166–208. 10.1016/0013-9351(69)90036-X (1969). 5788907 10.1016/0013-9351(69)90036-x

[37] Sadler, T. D., Rom, W. N., Lyon, J. L. & Mason, J. O. The use of asbestos-cement pipe for public water supply and the incidence of cancer in selected communities in Utah. *J. Community Health* **9**, 285–293. 10.1007/BF01338728 (1984). 6480892 10.1007/BF01338728

[38] Kusiorowski, R., Zaremba, T., Piotrowski, J. & Gerle, A. Thermal decomposition of asbestos-containing materials. *J. Therm. Anal. Calorim.* **113**, 179–188. 10.1007/s10973-013-3038-y (2013).

[39] Kusiorowski, R., Gerle, A., Kujawa, M., Śliwa, A. & Adamek, J. Characterisation of asbestos-containing wastes by thermal analysis. *J. Therm. Anal. Calorim.* **149**, 10681–10694. 10.1007/s10973-024-13312-3 (2024).

[40] Stutzman, P. E., Feng, P. & Bullard, J. W. Phase analysis of portland cement by combined quantitative X-ray powder diffraction and scanning electron microscopy. *J. Res. Natl. Inst. Stand. Technol.* **121**, 47. 10.6028/jres.121.004 (2016). 34434615 10.6028/jres.121.004 PMC7339643

[41] Wiktor, V., Grosseau, P., Guyonnet, R., Garcia-Diaz, E. & Lors, C. Accelerated weathering of cementitious matrix for the development of an accelerated laboratory test of biodeterioration. *Mater. Struct.* **44**, 623–640. 10.1617/s11527-010-9653-1 (2011).

[42] Aryal, A. & Morley, C. Mitigation of contamination and health risk: Asbestos management and regulatory practices. *Sustainability* **16**, 9740. 10.3390/su16229740 (2024).

[43] Emmett, E. A. Asbestos in high-risk communities: Public health implications. *Int. J. Environ. Res. Public Health* **18**, 1579. 10.3390/ijerph18041579 (2021). 33562413 10.3390/ijerph18041579 PMC7915393

[44] Vincenten, J., George, F., Martuzzi, M., Schröder-Bäck, P. & Paunovic, E. Barriers and facilitators to the elimination of asbestos related diseases—stakeholders’ perspectives. *Int. J. Environ. Res. Public Health* **14**, 1269. 10.3390/ijerph14101269 (2017). 29065497 10.3390/ijerph14101269 PMC5664770

[45] Bódizs, D. Az egészségügyi hulladékok COVID-19 pandémia következtében előálló környezetbiztonsági- és egészségügyi aspektusai (2022).

[46] Kusiorowski, R., Lipowska, B., Kujawa, M. & Gerle, A. Problem of asbestos-containing wastes in Poland. *Clean. Waste Syst.* **4**, 100085. 10.1016/j.clwas.2023.100085 (2023).

[47] European Economic and Social Committee. Opinion of the European Economic and Social Committee on ‘Freeing the EU from asbestos’ (2015).

[48] Romero Barriuso, A., Villena Escribano, B. M., González García, M. N., Segarra Cañamares, M. & Rodríguez Sáiz, A. Freeing the European Union from ASBESTOS (2018). (2032).

[49] Grammatikos, S. et al. On the mechanical recycling of decommisioned insulation polymer composite components. In *IOP Conference Series: Materials Science and Engineering* Vol. 842, 012002. 10.1088/1757-899X/842/1/012002 (2020).

[50] Siddique, R. Utilization of industrial by-products in concrete. *Procedia Eng.* **95**, 335–347. 10.1016/j.proeng.2014.12.192 (2014).

[51] Frank, L. & Joshi, A. The global spread of asbestos. *Ann. Glob. Health* **80**, 257. 10.1016/j.aogh.2014.09.016 (2014). 25459326 10.1016/j.aogh.2014.09.016

[52] Marsili, D. et al. Prevention of asbestos-related disease in countries currently using asbestos. *Int. J. Environ. Res. Public Health* **13**, 494. 10.3390/ijerph13050494 (2016). 27187433 10.3390/ijerph13050494 PMC4881119

[53] Visonà, S. D. et al. Impact of asbestos on public health: A retrospective study on a series of subjects with occupational and non-occupational exposure to asbestos during the activity of fibronit plant (Broni, Italy). *J. Public Health Res.*. 10.4081/jphr.2018.1519 (2018). 10.4081/jphr.2018.1519 PMC6321947 30687679

[54] Della Ventura, G. et al. Infra red spectroscopy of the regulated asbestos amphiboles. *Minerals* **8**, 413. 10.3390/min8090413 (2018). 31223499 10.3390/min8090413 PMC6586437

[55] Zholobenko, V., Rutten, F., Zholobenko, A. & Holmes, A. In situ spectroscopic identification of the six types of asbestos. *J. Hazard. Mater.* **403**, 123951. 10.1016/j.jhazmat.2020.123951 (2021). 33264995 10.1016/j.jhazmat.2020.123951

[56] Stefano, L., De, Cioffi, R. & Colangelo, F. Comparison between two FT-IR spectroscopy analytical procedures for micrograms determination of asbestos species in bulk materials. *Am. J. Anal. Chem.* **03**, 1–5. 10.4236/ajac.2012.31001 (2012).

[57] Hofko, B., Alavi, M. Z., Grothe, H., Jones, D. & Harvey, J. Repeatability and sensitivity of FTIR ATR spectral analysis methods for bituminous binders. *Mater. Struct.* **50**, 187. 10.1617/s11527-017-1059-x (2017).

[58] Lavine, B., Almirall, J., Muehlethaler, C., Neumann, C. & Workman, J. Criteria for comparing infrared spectra—A review of the forensic and analytical chemistry literature. *Forensic Chem.* **18**, 100224. 10.1016/j.forc.2020.100224 (2020).

[59] Bonnal, T. et al. How to determine the complex refractive index from infrared reflectance spectroscopy? *SN Appl. Sci.* **2**, 2070. 10.1007/s42452-020-03869-7 (2020).

[60] Obmiński, A. Asbestos cement products and their impact on soil contamination in relation to various sources of anthropogenic and natural asbestos pollution. *Sci. Total Environ.* **848**, 157275. 10.1016/j.scitotenv.2022.157275 (2022). 35905955 10.1016/j.scitotenv.2022.157275

[61] Tuzhikov, M. O., Tertishnikov, I. V. & Azarov, D. V. Study of urban atmosphere harmful substances adsorption into cement. *Procedia Eng.* **150**, 1531–1535. 10.1016/j.proeng.2016.07.105 (2016).

[62] Elfaleh, I. et al. A comprehensive review of natural fibers and their composites: An eco-friendly alternative to conventional materials. *Res. Eng.* **19**, 101271. 10.1016/j.rineng.2023.101271 (2023).

[63] Campopiano, A. et al. Risk assessment of the decay of asbestos cement roofs. *Ann. Occup. Hyg.* **53**, 627–638. 10.1093/annhyg/mep036 (2009). 19491148 10.1093/annhyg/mep036

[64] Chemrouk, M. & Attari, N. Durability of concrete with particular reference to high & performance concrete. In *Role of Concrete in Sustainable Development* 245–254 (Thomas Telford Publishing, 2003). 10.1680/rocisd.32477.0024.

[65] Giacobbe, C. et al. Spectroscopic study of the product of thermal transformation of chrysotile-asbestos containing materials (ACM). *Eur. J. Mineral.* **22**, 535–546. 10.1127/0935-1221/2010/0022-2038 (2010).

[66] Gil, L. K. T., Valdelamar Martínez, D., Franco, K. B., Arrieta Pastrana, A. & Saba, M. Mapping roof coverings of asbestos-cement, the first step to control the technical condition/threat and establish priorities for replacement in developing countries. *Heliyon* **10**, e37522. 10.1016/j.heliyon.2024.e37522 (2024). 39296010 10.1016/j.heliyon.2024.e37522 PMC11409103

[67] Punurai, W. & Davis, P. Prediction of asbestos cement water pipe aging and pipe prioritization using Monte Carlo simulation. *Eng. J.* **21**, 1–13. 10.4186/ej.2017.21.2.1 (2017).

[68] Mapar, M. et al. A composite index for sustainability assessment of health, safety and environmental performance in municipalities of megacities. *Sustain. Cities Soc.* **60**, 102164. 10.1016/j.scs.2020.102164 (2020).

[69] Papageorgiou, A. et al. Mapping and assessing indicator-based frameworks for monitoring circular economy development at the city-level. *Sustain. Cities Soc.* **75**, 103378. 10.1016/j.scs.2021.103378 (2021).

[70] Friederich, J. & Lazarova-Molnar, S. Reliability assessment of manufacturing systems: A comprehensive overview, challenges and opportunities. *J. Manuf. Syst.* **72**, 38–58. 10.1016/j.jmsy.2023.11.001 (2024).

[71] Zhou, Y., Zhu, S. & He, C. How do environmental regulations affect industrial dynamics? Evidence from China’s pollution-intensive industries. *Habitat Int.* **60**, 10–18. 10.1016/j.habitatint.2016.12.002 (2017).

[72] Sala, S., Ciuffo, B. & Nijkamp, P. A systemic framework for sustainability assessment. *Ecol. Econ.* **119**, 314–325. 10.1016/j.ecolecon.2015.09.015 (2015).

[73] Olawade, D. B. et al. Artificial intelligence in environmental monitoring: Advancements, challenges, and future directions. *Hygiene Environ. Health Adv.* **12**, 100114. 10.1016/j.heha.2024.100114 (2024).

